# Ultrasound Participated Optomechanical Frequency Comb Generation and Tunability in High‐Q Microbubble Cavities

**DOI:** 10.1002/nap2.70227

**Published:** 2026-08-03

**Authors:** Yuxing Zhang, Xing Niu, Hailin Chen, Sheng Liu, Lei Xu, Liying Liu

**Affiliations:** ^1^ Key Lab for Micro and Nanophotonic Structures (Ministry of Education, China) College of Future Information Technology Fudan University Shanghai China

**Keywords:** cavity optomechanics, microbubble resonator, optomechanical frequency comb, temperature sensing

## Abstract

Optomechanical frequency combs (OMFCs) are generated in high‐Q microbubble cavities when an optomechanical system is simultaneously driven by ultrasound and laser light. The combs originate from nonlinear interactions between optomechanical modes and ultrasonically driven mechanical oscillations. The comb spacing and intensity are manipulated by the frequency and amplitude of the ultrasound, as well as by the optical pump power and frequency detuning. The combination of the OMFC and the optical spring effect further enhances the temperature‐sensing sensitivity.

## Graphical abstract

Optomechanical frequency combs are generated in high‐Q microbubble cavities by simultaneous ultrasound and laser excitation, arising from nonlinear coupling between optomechanical modes and ultrasonically driven mechanical oscillations. Comb spacing and intensity are tunable through acoustic and optical parameters, whereas the optical spring effect amplifies temperature‐dependent shifts, enabling enhanced sensing.

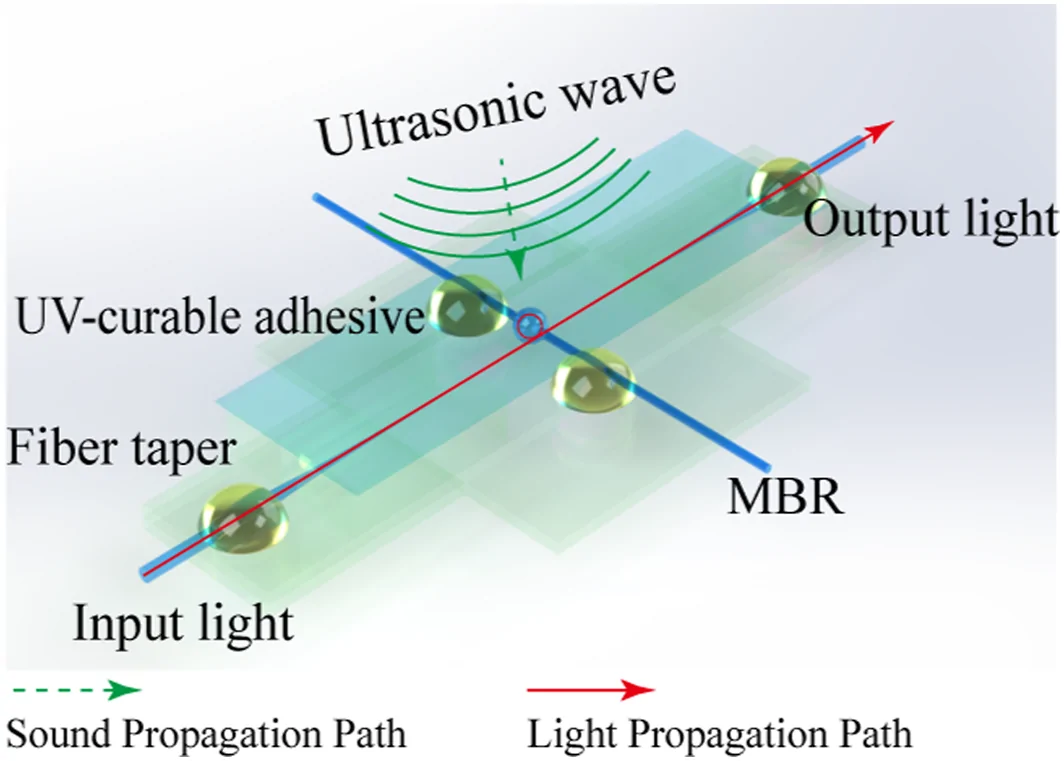

## 1 Introduction

A frequency comb is a series of equally spaced sharp frequency lines. Optical frequency comb with GHz‐level repetition rates attracts significant attention because it is a powerful light source in applications such as wide‐range metrology [1] and sensing [2, 3]. Microwave frequency combs offer enhanced stability and compatibility with electronic systems, but their limited spectral coverage and relatively low tunability hinder applications in broadband and hybrid photonic systems [4, 5, 6]. Optomechanical resonances generated in high‐Q optical microcavities are capable of being tuned spectrally from Hz to THz [7, 8], therefore optomechanical systems offer an ideal platform for realizing frequency combs that connect optical and microwave domains.

Unlike traditional optical combs or purely electrically driven radio‐frequency mechanical combs, the OMFC studied here is generated by optomechanical modulation of the optical whispering‐gallery mode and is detected as radio‐frequency beat notes after photodetection. It is initiated by pump‐induced self‐sustained oscillation of optomechanical modes [9, 10], which can trigger cascaded excitation and frequency mixing among multiple mechanical oscillations through intrinsic nonlinearities or external periodic modulation. However, intrinsic microcavity nonlinearities are constrained by specific cavity structures [11], material parameters [12], and stringent low‐temperature [13] or vacuum conditions [14], which limit the tunability and scalability of OMFC. External periodic modulation provides an effective alternative approach to achieve the generation of nonlinear mechanical waves such as sum‐frequency and difference‐frequency waves. Typical implementations, such as acoustic–optic modulators and ultrasonic transducers, have been applied in frequency locking [15, 16, 17] and highly sensitive acoustic detection [18, 19]. Nevertheless, the limited acousto–optic coupling strength still hinders the generation of a clear OMFC.

In this paper, we report OMFC generation driven by external ultrasound. An ultrasonic transducer effectively injects acoustic energy into a microbubble resonator (MBR), exciting its mechanical oscillations at the driving ultrasonic frequency. When the ultrasonically excited oscillations are strongly coupled to the optomechanical resonance through mechanical interaction, cascaded difference and sum frequency processes among the coupled mechanical oscillations give rise to an OMFC with kHz‐level spacing in the MHz frequency regime. Experimental results show that the cavity detuning and ultrasonic driving frequency effectively modulate the spacing of the frequency comb lines, whereas the pump power and ultrasonic amplitude determine the comb intensity, enabling multidimensional tunability of the optomechanical comb. In addition, temperature‐sensing measurements confirm the sensitivity enhancement from cascaded interactions among multiple comb lines, with higher‐order comb lines exhibiting several‐fold improvement compared with the fundamental optomechanical mode.

## 2 Experimental Setup

The experimental setup for realizing the ultrasonically driven OMFC using an MBR is shown in Figure 1. A tunable single frequency near‐infrared laser (CTL1550, TOPTICA Photonics AG) was used as the pump source. The intensity and polarization of the laser were determined by the optical attenuator and the fiber polarization controller, respectively. After passing through a beam splitter, 99% of the optical power was coupled into the MBR via a tapered fiber to ensure efficient evanescent coupling, whereas the remaining 1% was directed to a power meter for real‐time power monitoring. The transmitted laser output from the MBR was attenuated again and collected by a high‐bandwidth infrared photodetector (PDA10CF‐EC, Thorlabs). Then, a digital oscilloscope (TDS3032, Tektronix) and a spectrum analyzer (RSA306B, Tektronix) were connected via coaxial cables to acquire the optical resonance spectrum and power spectral density (PSD), respectively.

**FIGURE 1:**
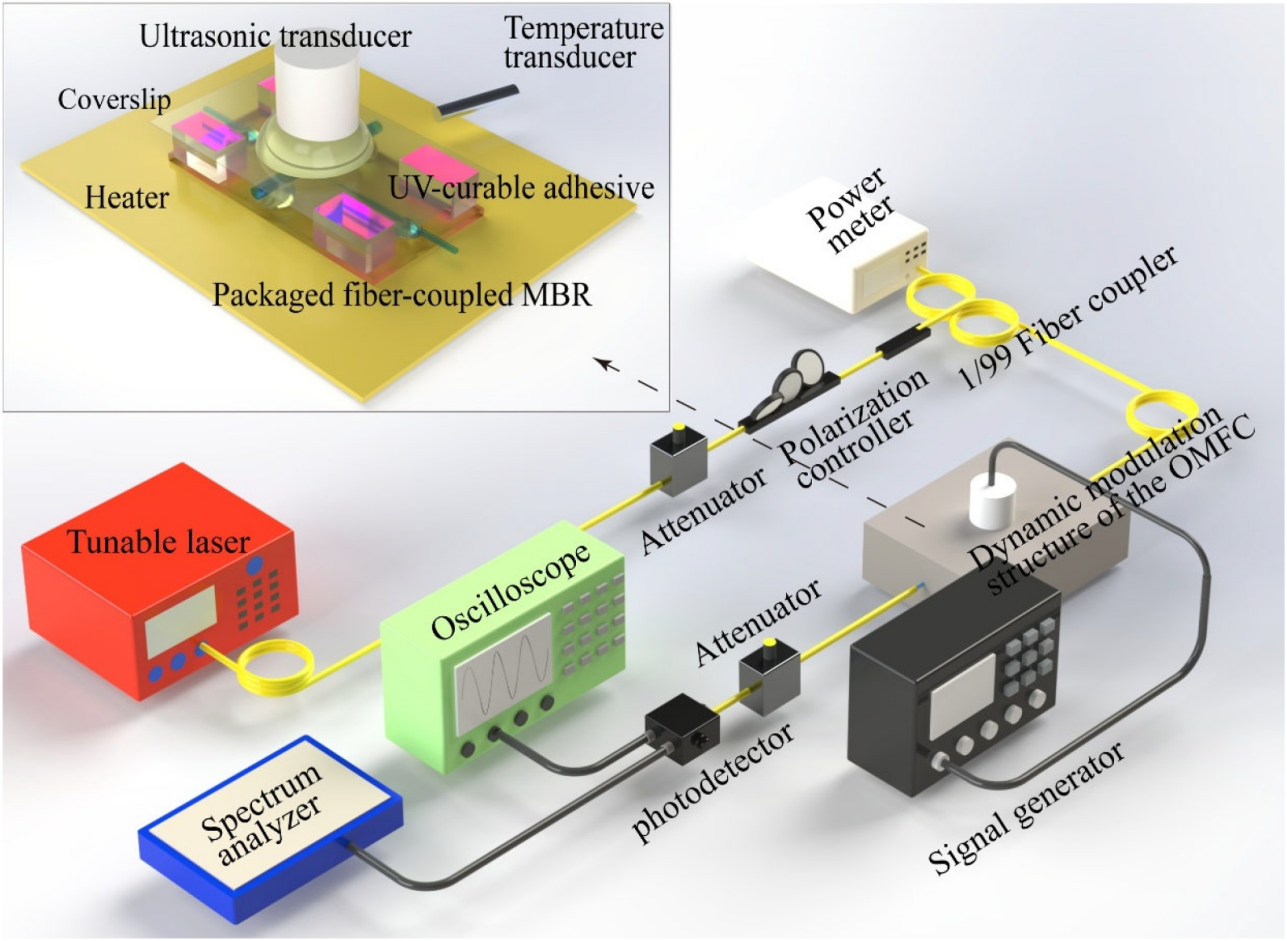
Experimental setup for ultrasonic modulation of the OMFC in the MBR. The inset shows the detailed structure of the dynamic modulation of the OMFC.

The inset of Figure 1 shows the detailed structure of the dynamic modulation of the OMFC. An ultrasonic transducer, driven by a signal generator, generates acoustic waves that are efficiently transferred to the MBR. The transducer is rigidly bonded to a cover glass using NOA 81 UV‐curable adhesive, forming a mechanically integrated optomechanical and acoustic platform. In addition, a temperature control unit is used to perform temperature sensing experiments of the OMFC. The ultrasonic transducer operates over a 0.5–10 MHz frequency range, with its effective bandwidth determined by the intrinsic mechanical response of the device. The driving amplitude can be adjusted from 0 to 10 Vpp.

To establish a geometrical model for the finite‐element simulations, the inner and outer contours of the microbubble resonator were extracted from the microscope image, as shown in Figure 2. The fabricated MBR had a diameter of approximately 305 μm and a wall thickness of approximately 10 μm. The inner and outer bubble profiles were obtained from Gaussian fitting under the assumption of nonvariation of total volume [20]:

(1) $$r_{\text{out}/\text{in}}\left(z\right)=R_{\text{out}/\text{in}}+A_{\text{out}/\text{in}}\;\exp\;\left(-\alpha_{\text{out}/\text{in}}z^{2}\right)$$

in which R_ is the radius of the capillaries on each side of the MBR, R_+A_ is the equatorial radius of the MBR, α_ is the fitting parameter related to the curvature along the *z* axis of contour. The subscripts ‘*out*’ and ‘*in*’ denotes the outer and inner contour of the MBR, respectively. Gaussian fitting gave *r*_*in*_ = 48.92 + 97.25 exp (−5.46 × 10‐5z^2^) and *r*_out_ = 71.41 + 81.28 exp (−4.63 × 10‐5z^2^), respectively.

**FIGURE 2:**
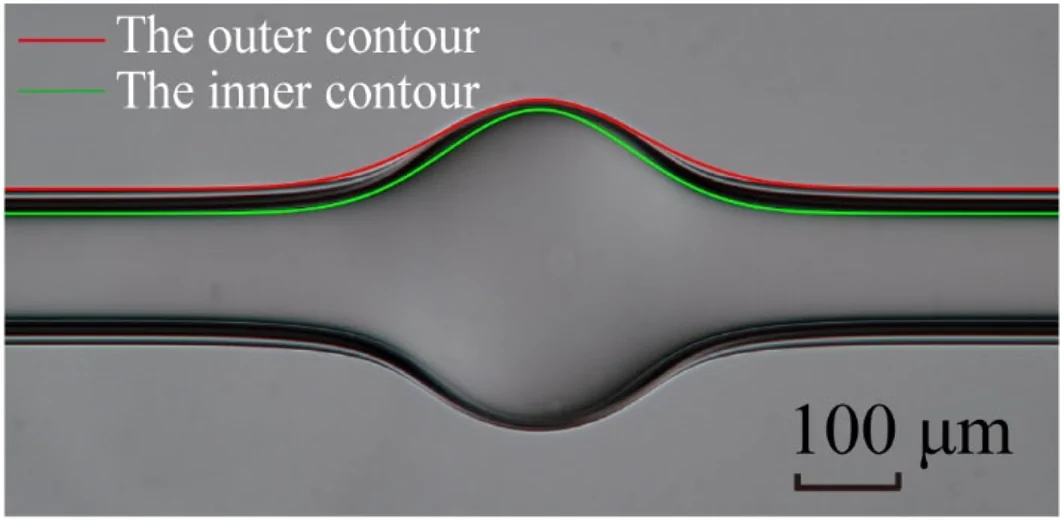
Results of surface contour fitting for the MBR.

## 3 The Generation Method of OMFC

The structure of the packaged fiber‐coupled MBR is shown in Figure 3a. The tapered fiber was positioned near the equatorial region of the MBR to excite the whispering‐gallery modes through evanescent coupling and was subsequently fixed using UV‐curable adhesive to improve mechanical stability. In this packaged structure, the ultrasonic transducer was bonded to the cover glass using UV‐curable adhesive, allowing the acoustic wave to propagate through the cover glass and couple into the MBR. When a pump laser with 12.62 mW at 1532.772 nm was applied, self‐sustained optomechanical oscillations at 7.960 MHz together with its higher‐order harmonics were generated. Furthermore, a low frequency optomechanical mode at 553 kHz was observed, as shown in Figure 3b. This kHz low frequency mode arose from the global oscillation of the microbubble‐capillary structure, whereas the MHz‐range oscillation was mainly localized to the bubble region. The spatial distributions of these oscillations are confirmed by the finite element simulations, as shown in Figure 3c. Such coexistence of global and localized optomechanical oscillations has also been reported previously [21]. The low frequency mode couples with the 7.960 MHz optomechanical mode, giving rise to cascaded sidebands spaced by 553 kHz around the 7.960 MHz resonance.

**FIGURE 3:**
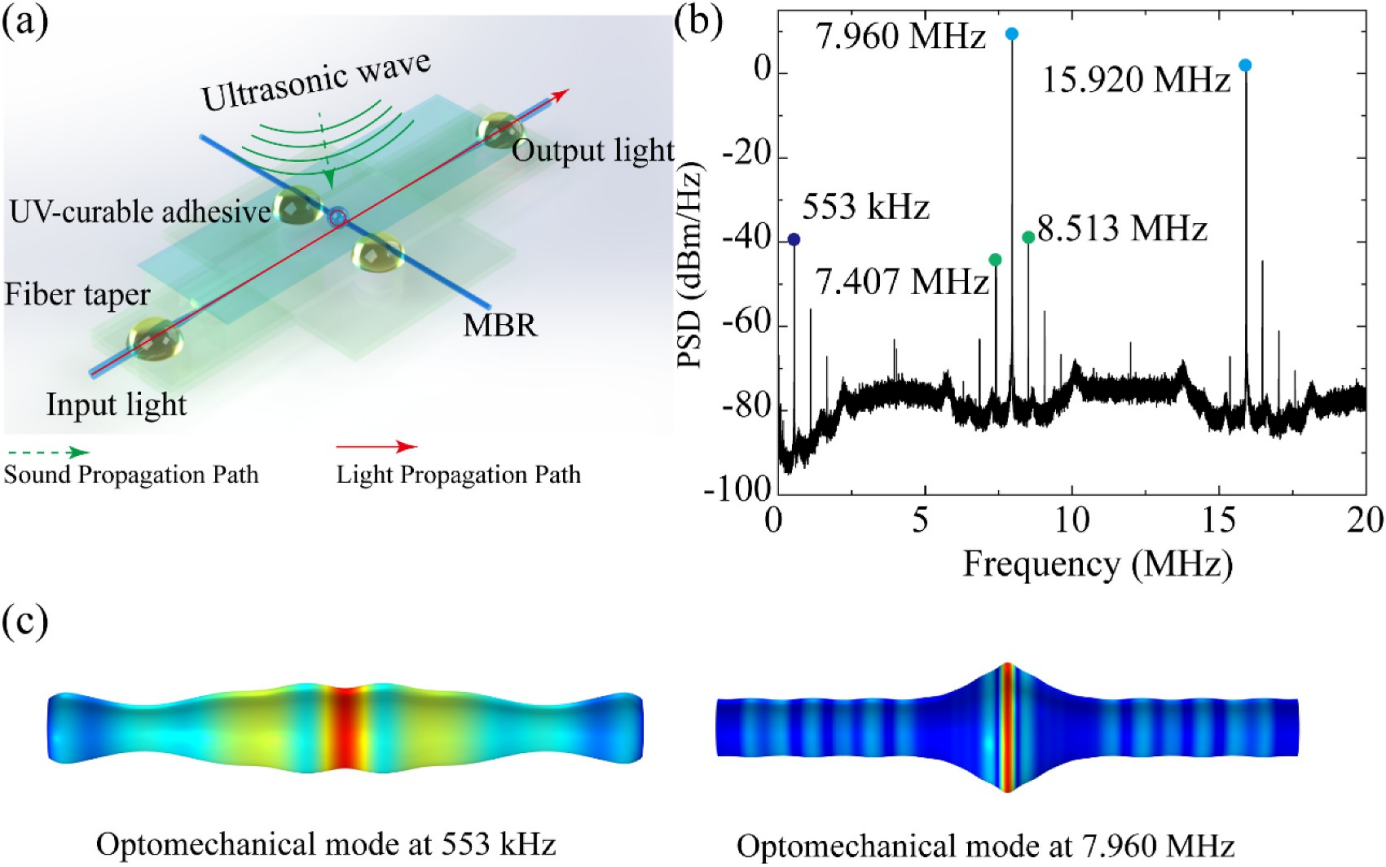
(a) Structure of the packaged fiber‐coupled MBR. (b) Measured PSD of the optomechanical oscillation. (c) Three dimensional displacement distributions of the optomechanical modes at frequencies of 553 kHz and 7.960 MHz.

A pronounced OMFC was generated, as shown in Figure 4a, when an ultrasonic wave of 4.021 MHz and 4.0 Vpp was applied to the MBR to drive the acoustic–mechanical coupling. Under this excitation, the optomechanical resonance slightly shifted to 7.968 MHz, accompanied by the appearance of higher‐order harmonics. Within the 7–9 MHz range, 25 uniformly spaced comb lines were observed (see Figure 4b), whereas the corresponding difference‐frequency comb components between ultrasound and optomechanical resonance appeared in the 0–1 MHz range, as shown in Figure 4c. Figure 4d further confirms that the comb exhibited strictly linear spacing, with a fundamental spacing of 74 kHz. The formation of the comb arose from coherent nonlinear mixing between the intrinsic optomechanical mode (*Ω*_1_) and the ultrasonically induced mechanical oscillation (*Ω*_2_). The comb spacing between the two oscillations, Δ*Ω* = 2*Ω*_2_ −*Ω*_1_ ≈ 74 kHz, modulated the intracavity optical field, giving rise to a series of equally spaced sidebands at *Ω*_1_ ± nΔ*Ω*. On the other hand, output of the ultrasonic power at 8.042 MHz (2*Ω*_2_) was too low to support comb generation.

**FIGURE 4:**
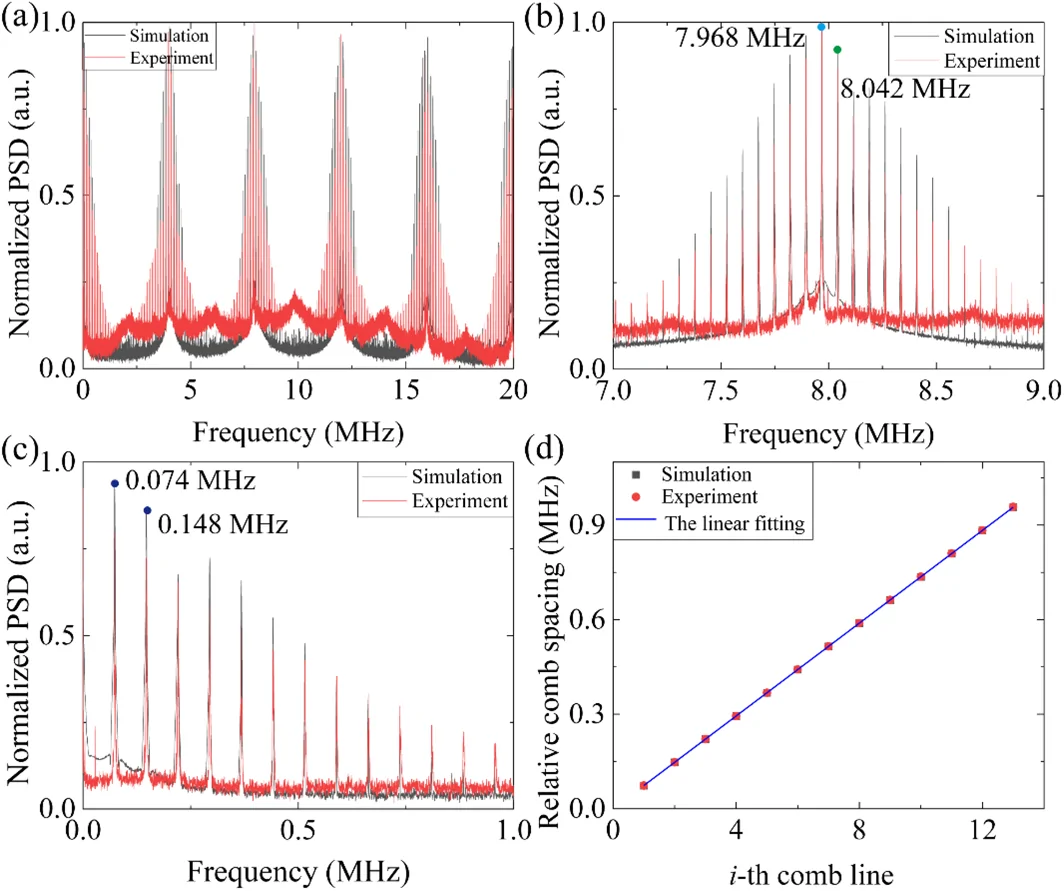
Experimental and simulated spectra of the ultrasonically induced OMFC. (a) Broadband power spectral density in the 0–20 MHz range. (b) The enlarged PSD around 7.968 MHz optomechanical mode. Uniformly spaced sidebands can be clearly observed. Blue dots represent the optomechanical resonance, green dots represent the ultrasonically induced mechanical oscillation. (c) Low frequency components showing the 74 kHz difference‐frequency signal and harmonics. (d) Relative spacing between the first comb line and the *i*th comb line versus the comb order in the 0–1 MHz range.

## 4 Theory

The ultrasonic coupling induced formation of the OMFC is realized in a MBR. In addition to the intrinsic radiation pressure induced interaction, the externally applied ultrasound introduces periodic mechanical oscillations that further modulate the optical modes, resulting in three‐wave coupling among the ultrasonic field, optomechanical mode, and optical mode. To illustrate this mechanism, the system dynamics are described by the classical optomechanical motional equations as follows:

(2) $$\frac{da}{dt}=\left(i\left(\Delta \omega +G_{1}x_{1}+G_{2}x_{2}\right)-\frac{\kappa }{2}\right)a+\sqrt{\kappa_{ex}}a_{in}$$

(3) $$\frac{d^{2}x_{1}}{dt^{2}}+\Gamma_{1}\frac{dx_{1}}{dt}+\Omega_{1}^{2}x_{1}=\frac{\hbar G_{1}{\left|a\right|}^{2}+F_{us}\left(t\right)}{m_{eff1}}$$

(4) $$\frac{d^{2}x_{2}}{dt^{2}}+\Gamma_{2}\frac{dx_{2}}{dt}+\Omega_{2}^{2}x_{2}=\frac{\hbar G_{2}{\left|a\right|}^{2}+F_{us}\left(t\right)}{m_{eff2}}$$

Where *a* is the intracavity optical field amplitude; *a*_*in*_ is the amplitude of the input optical field; Δ*ω* is the frequency detuning between the pump laser and the optical resonance of the microcavity; *κ* is the total optical loss rate of the cavity; *Ω*_1,2_ are the frequencies of the optomechanical mode and the ultrasonically induced mechanical oscillation, respectively; *G*_1,2_ are the mechanical coupling coefficient of mechanical oscillation 1 and 2, respectively; *κ*_*ex*_ is the external coupling loss rate; *Γ*_1,2_ are the damping rates of mechanical oscillation 1 and 2, respectively; and m_*eff*1,2_ are the corresponding effective masses of mechanical oscillation 1 and 2, respectively; *F*_*us*_ is the periodic oscillatory force exerted on the microcavity by the ultrasound, which can be expressed as:

(5) $$F_{us}=k_{t}\cdot V_{pp}\cdot \sin\left(\Omega_{2}t\right)$$

Where *k*_*t*_ is the transduction efficiency of the ultrasonic transducer applied to the microcavity, *V*_*pp*_ is the peak‐to‐peak voltage of the signal applied to the ultrasonic transducer by the function generator. Finally, based on the input–output relation $a_{out}=a_{in}-\sqrt{\kappa_{ex}}a$, the temporal evolution of the output optical field can be obtained, and its spectral characteristics are further derived by Fourier transformation.

Numerical simulations were performed using experimental parameters, that is *λ* = 1531.772 nm, *Q* = 1.02 × 10^7^, *Ω*_1_ = 7.9675 MHz, *Ω*_2_ = 4.021 MHz, *Γ*_1_ = 1.0 × 10^3^ Hz, *Γ*_2_ = 1.0 × 10^4^ Hz, and coupling coefficient *G*_1_ = 1.0 × 10^18^ Hz/m and *G*_2_ = 1.0 × 10^17^ Hz/m. The system was driven by an external acoustic force with a transduction efficiency *k*_*t*_ = 1.2 × 10^−5^ N/V. The simulated spectra reproduce the experimentally observed frequency shift, harmonic generation, and the sideband spacing of approximately 74 kHz, as shown in Figure 4a–d. These results confirm that the observed comb is a direct consequence of nonlinear mixing described by Equations ((2), (3), (4)).

## 5 Results and Discussions

### 5.1 Relation of OMFC on Ultrasound Parameters

Figure 5a shows the evolution of the OMFC distribution under different ultrasonic frequencies. The black dots represent the optomechanical mode frequency, the red dots represent the ultrasound frequency. When 2*Ω*_2_ goes closer to the optomechanical mode resonant frequency, comb spacing becomes less and more lines emerge. When ultrasound frequency reaches 3.982 MHz at which 2*Ω*_2_ ≈ *Ω*_1_, a stable injection locking state was achieved, that is the optomechanical mode resonant frequency is dragged to the ultrasound frequency [15, 16, 17]. The sideband lines at *Ω*_2_ = 3.982 MHz come from the difference frequency between locked mechanical oscillation and the low frequency mode (∼500 kHz). Comb appears again when ultrasound frequency increases further, the comb spacing is 93 kHz at *Ω*_2_ = 4.030 MHz. Figure 5b illustrates the dependence of optomechanical resonance frequency (black dots) with the ultrasound frequency (red dots). The dispersive shift of mechanical resonant frequency is clear evidence of injection locking. Figure 5c shows the change of the peak intensity of the resonant lines. In the locked regime, the amplitudes of both the ultrasonically induced mechanical oscillation and the optomechanical mode were simultaneously enhanced. Similar locking‐induced amplitude enhancement has been observed in optomechanical systems [17, 22]. The observed behavior may be associated with nonlinear injection locking in strongly coupled optomechanical systems.

**FIGURE 5:**
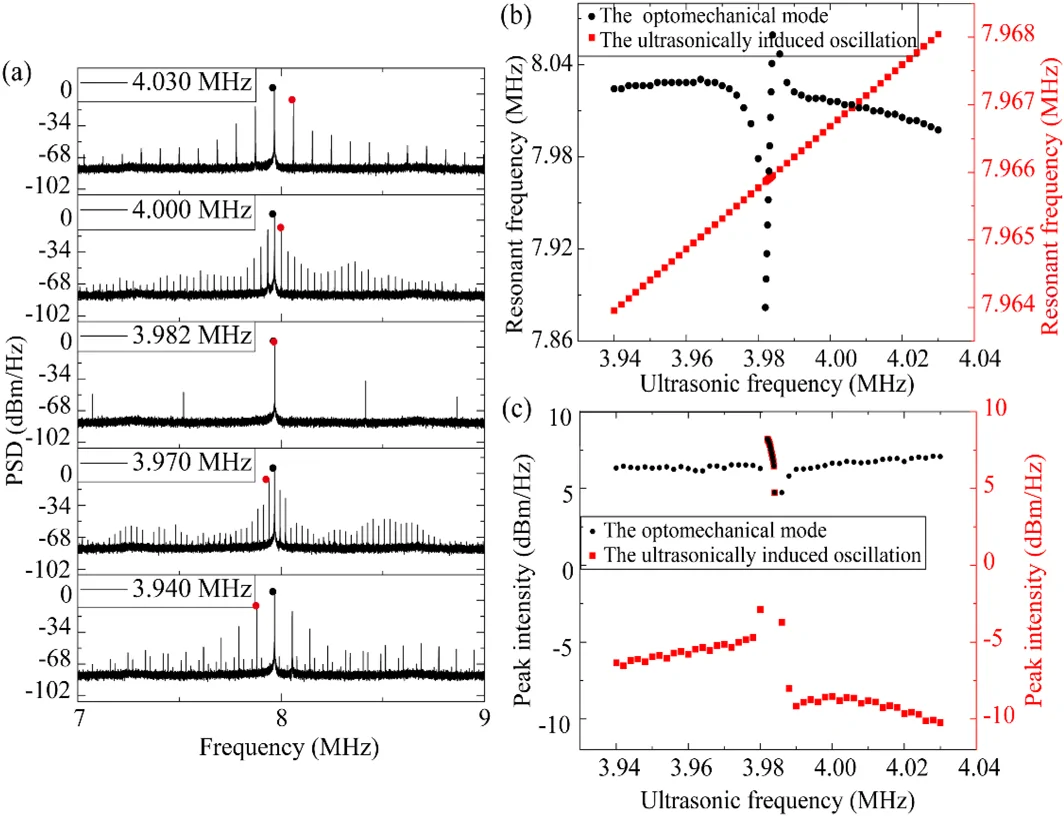
(a) Mechanical spectra of the OMFC at different ultrasonic frequencies. Black dots represent the optomechanical resonance, and red dots represent the ultrasonically induced mechanical oscillation. (b) Frequency evolution and (c) peak amplitudes of the optomechanical resonance and the ultrasonically induced mechanical oscillation versus the ultrasonic frequency.

We also investigated the relation of ultrasound driving amplitudes to the generation of OMFC. The ultrasound frequency was fixed at 4.010 MHz, and its amplitude was swept from 0 to 10.0 Vpp in steps of 0.5 Vpp. At low amplitude, only the fundamental optomechanical mode and a few weak sidebands are visible. Increasing the amplitude substantially enhances the comb strength, expands the number of sidebands, and improves the overall signal‐to‐noise ratio. The comb lines were symmetrically distributed on both sides of the fundamental optomechanical resonance, as shown in Figure 6a. Figure 6b shows that when ultrasound power increased, compared with the stable ultrasonically driven oscillation frequency, the optomechanical mode exhibited a typical optical spring response due to the thermal‐induced refractive‐index change as the ultrasound heats the cavity. During the 10 minutes amplitude sweep, an increase of approximately 0.2°C in the ambient temperature was measured, resulting in a slow drift of the optomechanical frequency. When the amplitude reached 9.0 Vpp, the environmental temperature rise is large enough such that the optical mode resonance shifts away from the laser frequency, thus the sustained mechanical oscillation is suppressed. Nevertheless, the experimental results clearly indicate that higher ultrasonic power strengthens the optomechanical coupling and generates higher‐order sidebands. Figure 6c presents the evolution of both the peak amplitude of the ultrasonically induced mechanical oscillation and the optomechanical mode. As the ultrasound driving amplitude increases, the efficiency of nonlinear frequency mixing is significantly enhanced, leading to increased energy transfer from the fundamental optomechanical oscillation into higher‐order comb lines. Consequently, the effective energy stored in the fundamental optomechanical mode is reduced, resulting in a decrease in its oscillation amplitude. On the other hand, the system maintains excellent comb stability at fixed ultrasonic amplitude, enabled by the thermally induced self‐stabilization of the microcavity [23], as shown in Figure 7a. The linear fitting of the comb lines, shown in Figure 7b, reveals an excellent linear relationship, confirming the robustness of the OMFC.

**FIGURE 6:**
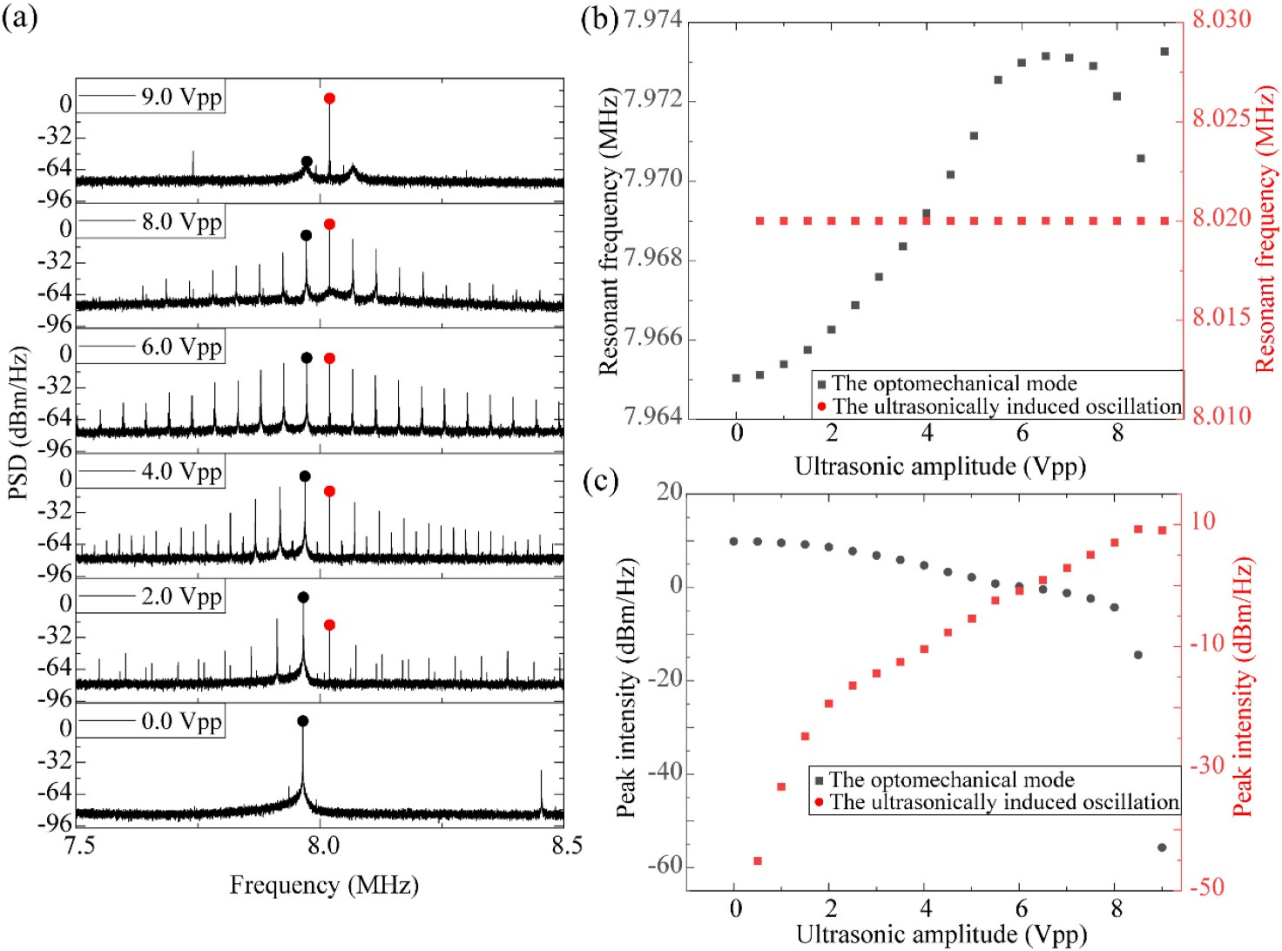
(a) Spectral distributions of the OMFC under different driving amplitudes. Black dots represent the optomechanical resonance, and red dots represent the ultrasonically induced mechanical oscillation. (b) Frequency evolution and (c) peak amplitudes of the optomechanical resonance and the ultrasonically induced mechanical oscillation versus the ultrasonic amplitude.

**FIGURE 7:**
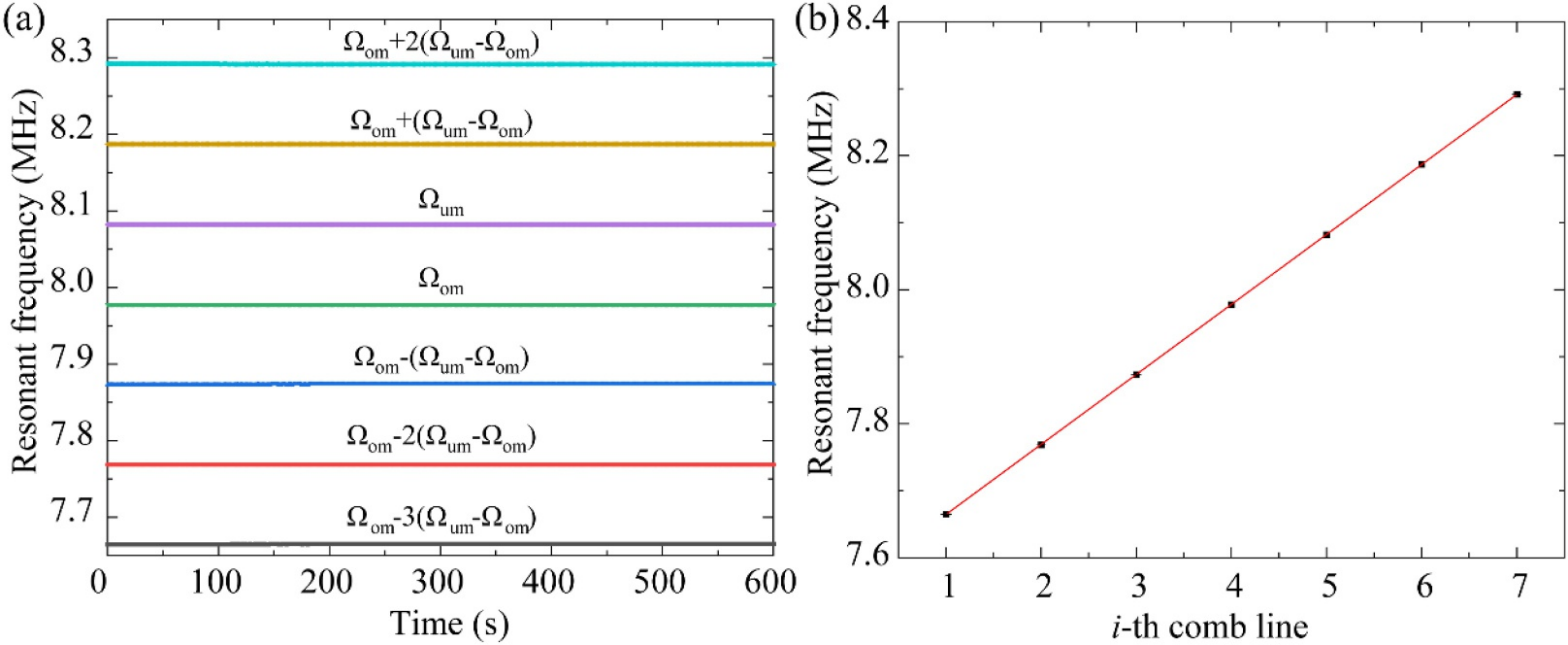
(a) 10 minutes stability measurement of the OMFC. (b) Linear fitting of the comb line frequencies.

### 5.2 Relation of OMFC on Pump Laser Parameters

We also explored systematically the influence of the pump laser frequency and optical power on the OMFC performance. Detuning of the laser frequency generates optical spring effect [24, 25], leading to a shift in optomechanical mode resonant frequency, thus enables the control of comb spacing and its spectral distribution. To observe the detuning influence, the pump laser was fixed at 12.62 mW, meanwhile the ultrasound driving was maintained at 4.021 MHz with an amplitude of 4.0 Vpp. As the laser wavelength was detuned from 1531.344 to 1531.350 nm in steps of 1 p.m., the corresponding OMFC spectra were recorded in Figure 8a. Figure 8b presents the corresponding shift of the optomechanical resonance frequency, which reflects a typical optical spring effect under blue detuning, at the same time the ultrasonically induced mechanical oscillation remains fixed. This phenomenon confirms that the spectral evolution of the comb is determined by the optical spring effect induced change of the optomechanical resonant frequency. Furthermore, Figure 8c reveals that the peak amplitudes of the optomechanical mode and the ultrasonically induced mechanical oscillation change in opposite way, arising from mode competition [16]. This behavior is consistent with the amplitude and frequency dependent modulation of the OMFC distribution, confirming the competitive energy redistribution between the two mechanical oscillations under varying driving conditions.

**FIGURE 8:**
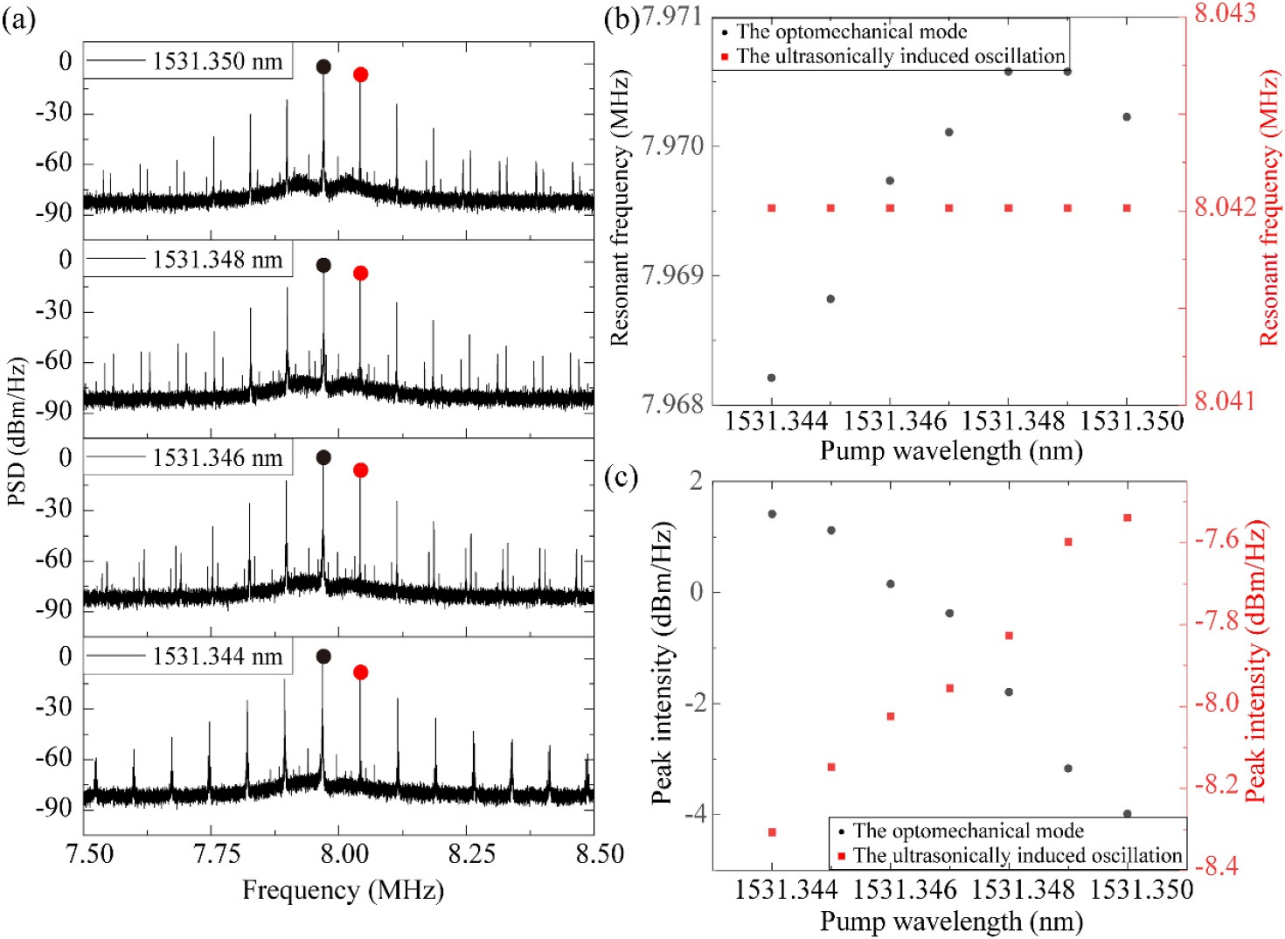
(a) Spectral distributions of the OMFC under different pump laser frequencies. Black dots represent the optomechanical resonance, and red dots represent the ultrasonically induced mechanical oscillation. (b) Frequency evolution and (c) peak amplitudes of the optomechanical resonance and the ultrasonically induced mechanical oscillation versus pump laser frequency.

We also investigated the influence of the pump laser power on the comb generation. With the pump wavelength fixed at 1531.350 nm and the ultrasonic drive maintained at 4.016 MHz and 4.0 Vpp, the laser power increased from 10 to 13 mW in steps of 0.5 mW, and the corresponding evolution of the OMFC was recorded, as shown in Figure 9a. Obvious comb starts to emerge when laser power is over 11 mW which is the self‐sustained oscillation threshold of the optomechanical mode. Meanwhile, Figure 9b shows that resonant frequency of the mode shifts when laser power increases, which can be contributed to the heating‐initiated thermal‐refractive index change. The increasing pump power drives the optomechanical mode into self‐sustained oscillation and gradually saturates, as shown in Figure 9c. Consequently, the enhanced optomechanical oscillation significantly improves the nonlinear frequency‐mixing and photo‐mechanical conversion efficiency, leading to an increase in the total mechanical oscillation power distributed among multiple frequency components. As a result, more and stronger comb lines appear.

**FIGURE 9:**
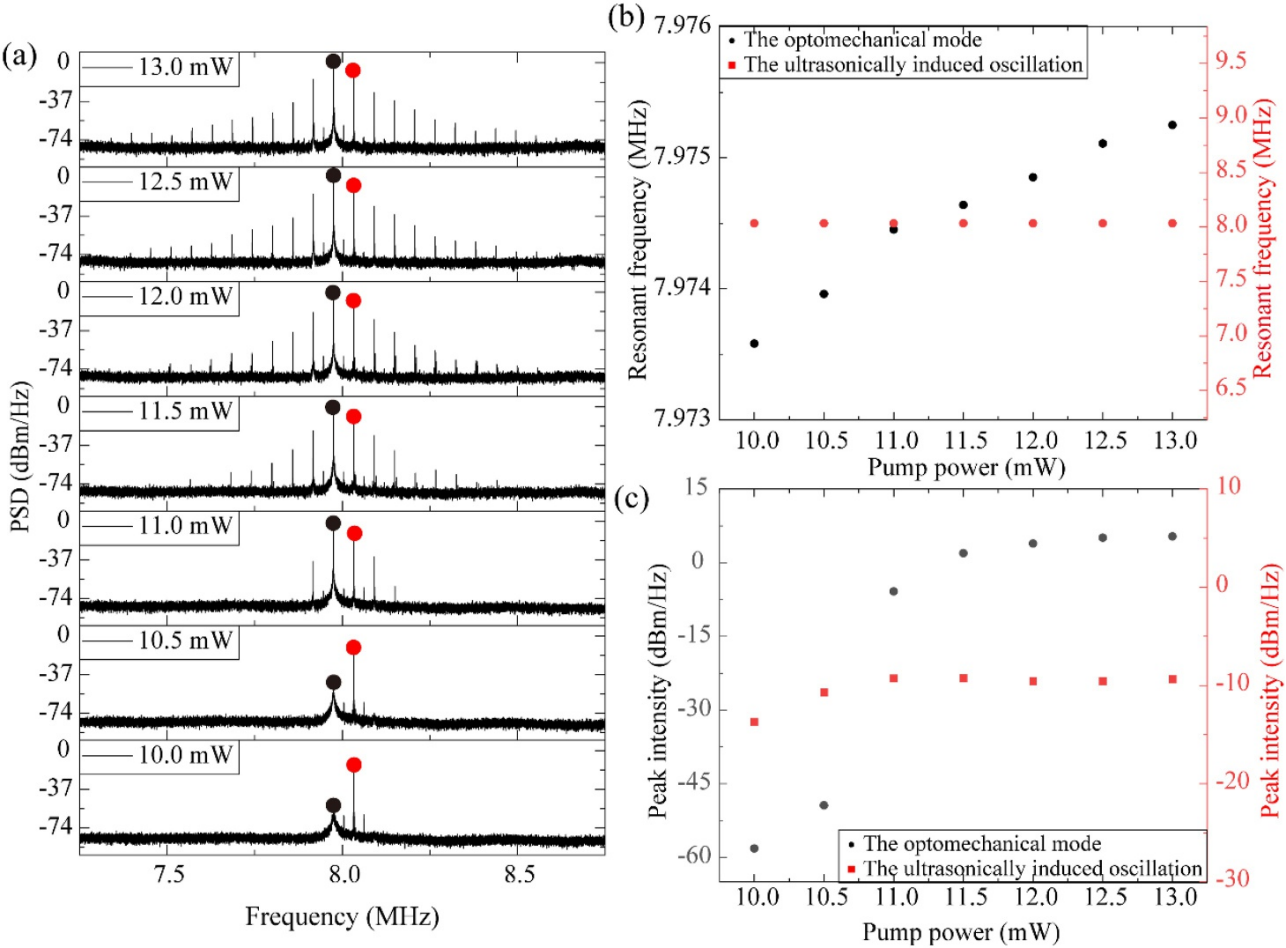
(a) Spectral distributions of the OMFC under different pump powers. Black dots represent the optomechanical resonance, and red dots represent the ultrasonically induced mechanical oscillation. (b) Frequency evolution and (c) peak amplitudes of the optomechanical resonance and the ultrasonically induced mechanical oscillation versus the pump power.

## 6 The Temperature Sensing of OMFC

OMFCs provide multiple uniformly spaced spectral lines, enabling temperature perturbations to be mapped simultaneously onto a series of comb lines and amplifying the measurable frequency shift. Since the ultrasonic frequency is essentially insensitive to temperature, the temperature dependence of the comb spacing is dominated by the optomechanical mode. As a result, the temperature sensitivity of the *n*th comb line follows:

(6) $$\frac{d\Omega_{n}}{dT}=n\frac{d\Omega_{1}}{dT}$$

Thus, temperature‐induced shift increases proportionally with the comb order, making higher‐order lines substantially more sensitive to small environmental perturbations.

Experimentally, the MBR was placed on a temperature‐controlled heating stage operated in a closed‐loop module to suppress environmental fluctuations. With the pump wavelength fixed at 1531.350 nm and power of 12.62 mW and the ultrasonic frequency was 4.018 MHz with an amplitude of 4.0 Vpp, the ambient temperature changed from 25.3°C to 25.8°C in steps of 0.05°C. The resulting evolution of the OMFC with temperature is shown in Figure 10a. Figure 10b presents the frequency change of the optomechanical mode together with two comb lines versus temperature. The frequency of the ultrasonically induced mechanical oscillation is also presented in comparison. The optomechanical mode exhibits a sensitivity of −7.82 kHz/°C, whereas the two comb lines show sensitivities of −46.78 kHz/°C and 31.27 kHz/°C, respectively, corresponding to six‐fold and four‐fold enhancement relative to the fundamental response and are consistent with the expected proportional scaling rule. The ultrasonically induced mechanical oscillation remains insensitive to temperature, enabling a clear comparison between the thermally responsive optomechanical comb and the temperature‐insensitive ultrasonic oscillation. Figure 10c illustrates the enhancement factor of temperature sensitivity as a function of comb line order, which is in good agreement with Equation (6). The theoretical temperature detection limit of the system is estimated to be 7.3 × 10^−6^ °C derived from real‐time spectrum analyzer (RSA306B, Tektronix) with a 1.18 Hz resolution and an associated frequency uncertainty of approximately 0.34 Hz. This performance represents approximately an order of magnitude improvement over previously reported optomechanical temperature sensing schemes [26, 27].

**FIGURE 10:**
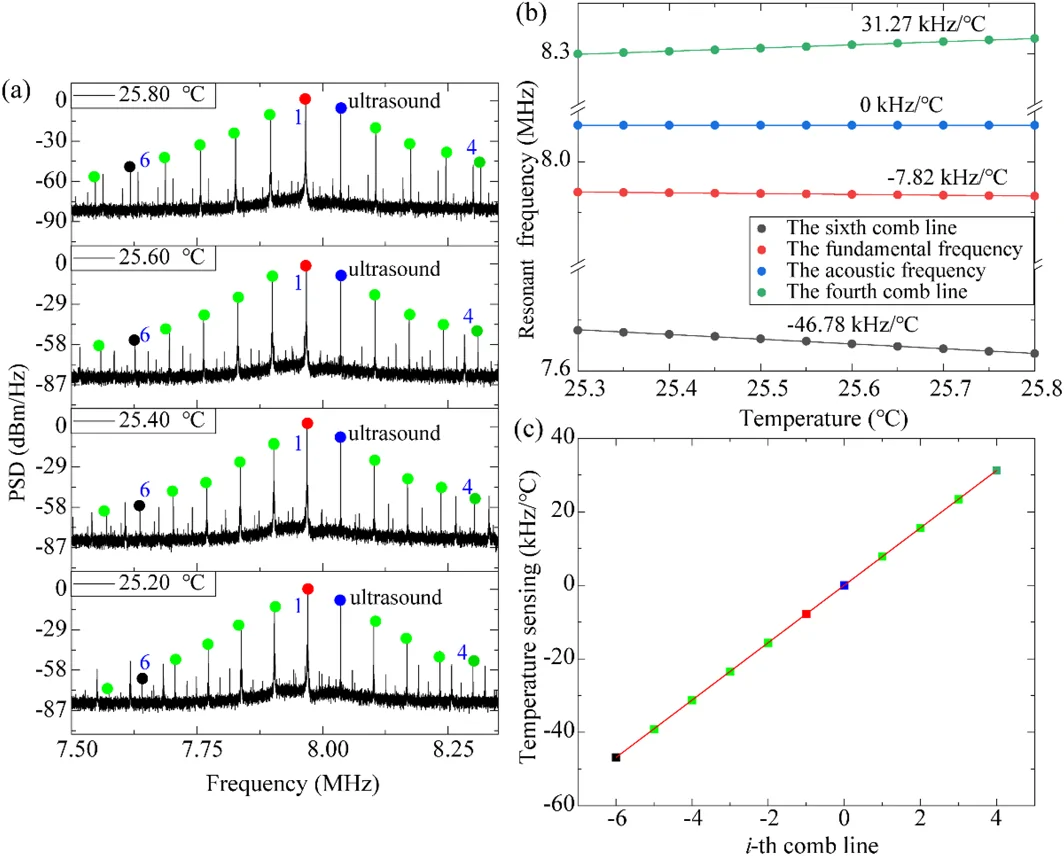
(a) Spectral distributions of the OMFC under different temperatures. (b) Temperature dependence of the fundamental optomechanical mode (red dots), the sixth‐order comb line (black dots), the fourth‐order comb line (green dots) and the ultrasonically induced mechanical oscillation (blue dots). (c) Relationship of the temperature sensitivity on comb line order.

## 7 Conclusion

In conclusion, optomechanical frequency combs are successfully generated in microbubble resonators when the cavity is driven both by laser and ultrasound. Comb line spacing depends on optical detuning and ultrasonic driving frequency, whereas comb strength depends on optical pump power and ultrasonic amplitude. The scaling rule of temperature sensitivity with comb line order is also confirmed, with an experimental detection limit of 7.3 × 10^−6^ °C, approximately an order of magnitude better than reported optomechanical temperature sensing results. Our results present a new way of efficiently generating optomechanical frequency combs and reveal their profound potential in high‐precision applications.

## Author Contributions

**Yuxing Zhang:** writing – original draft, investigation, validation, methodology, software, data curation, visualization, formal analysis, conceptualization. **Xing Niu:** methodology, software, investigation. **Hailin Chen:** methodology, investigation. **Sheng Liu:** methodology, conceptualization. **Lei Xu:** writing – review and editing, funding acquisition, resources, project administration, supervision. **Liying Liu:** funding acquisition, writing – review and editing, resources, project administration, supervision.

## Funding

This work was supported by the National Natural Science Foundation of China (NSFC) (Grants 11874122, 11474070, 61327008 and 11074051); Doctoral Program of Higher Education (Grant 20130071130004).

## Conflicts of Interest

The authors declare no conflicts of interest.

## Data Availability

The data that support the findings of this study are available from the corresponding author upon reasonable request.
